# Sample size calculation

**DOI:** 10.4103/0974-7788.59946

**Published:** 2010

**Authors:** Prashant Kadam, Supriya Bhalerao

**Affiliations:** *Department of Clinical Pharmacology, Seth GS Medical College and KEM Hospital, Parel, Mumbai - 400 012, India*; 1 *Department of Clinical Pharmacology, TNMC and BYL Nair Hospital, Mumbai Central, Mumbai - 400 008, India*

## INTRODUCTION

One of the pivotal aspects of planning a clinical study is the calculation of the sample size. It is naturally neither practical nor feasible to study the whole population in any study. Hence, a set of participants is selected from the population, which is less in number (size) but adequately represents the population from which it is drawn so that true inferences about the population can be made from the results obtained. This set of individuals is known as the “sample.”

In a statistical context, the “population” is defined as the complete set of people (e.g., Indians), the “target population” is a subset of individuals with specific clinical and demographic characteristics in whom you want to study your intervention (e.g., males, between ages 45 and 60, with blood pressure between 140 mmHg systolic and 90 mmHg diastolic), and “sample” is a further subset of the target population which we would like to include in the study. Thus a “sample” is a portion, piece, or segment that is representative of a whole.

## ATTRIBUTES OF A SAMPLE

- Every individual in the chosen population should have an equal chance to be included in the sample.
- Ideally, choice of one participant should not affect the chance of another's selection (hence we try to select the sample randomly – thus, it is important to note that random sampling does not describe the sample or its size as much as it describes how the sample is chosen).

The sample size, the topic of this article, is, simply put, the number of participants in a sample. It is a basic statistical principle with which we define the sample size before we start a clinical study so as to avoid bias in interpreting results. If we include very few subjects in a study, the results cannot be generalized to the population as this sample will not represent the size of the target population. Further, the study then may not be able to detect the difference between test groups, making the study unethical.

On the other hand, if we study more subjects than required, we put more individuals to the risk of the intervention, also making the study unethical, and waste precious resources, including the researchers’ time.

The calculation of an adequate sample size thus becomes crucial in any clinical study and is the process by which we calculate the optimum number of participants required to be able to arrive at ethically and scientifically valid results. This article describes the principles and methods used to calculate the sample size.

Generally, the sample size for any study depends on the:[1]

- Acceptable level of significance
- Power of the study
- Expected effect size
- Underlying event rate in the population
- Standard deviation in the population.

Some more factors that can be considered while calculating the final sample size include the expected drop-out rate, an unequal allocation ratio, and the objective and design of the study.[2]

## LEVEL OF SIGNIFICANCE

Everyone is familiar with the “*p*” value. This is the “level of significance” and prior to starting a study we set an acceptable value for this “*p*.” When we say, for example, we will accept a *p*<0.05 as significant, we mean that we are ready to accept that the probability that the result is observed due to chance (and NOT due to our intervention) is 5%. To put it in different words, we are willing to accept the detection of a difference 5 out of 100 times when actually no difference exists (i.e., get a “false positive” result). Conventionally, the *p* value of 5% (*p* = 0.05) or 1% (*p* = 0.01), which means 5% (or 1%) chance of erroneously reporting a significant effect is accepted.

## POWER

Sometimes, and exactly conversely, we may commit another type of error where we fail to detect a difference when actually there is a difference. This is called the Type II error that detects a false negative difference, as against the one mentioned above where we detect a false positive difference when no difference actually exists or the Type I error. We must decide what is the false negative rate we are willing to accept to make our study adequately powered to accept or reject our null hypothesis accurately.

This false negative rate is the proportion of positive instances that were erroneously reported as negative and is referred to in statistics by the letter β. The “power” of the study then is equal to (1 –β) and is the probability of failing to detect a difference when actually there is a difference. The power of a study increases as the chances of committing a Type II error decrease.

Usually most studies accept a power of 80%. This means that we are accepting that one in five times (that is 20%) we will miss a real difference. Sometimes for pivotal or large studies, the power is occasionally set at 90% to reduce to 10% the possibility of a “false negative” result.

## EXPECTED EFFECT SIZE

We can understand the concept of “effect size” from day-to-day examples. If the average weight loss following one diet program is 20 kg and following another is 10 kg, the absolute effect size would be 10 kg. Similarly, one can claim that a specific teaching activity brings about a 10% improvement in examination scores. Here 10 kg and 10% are indicators of the claimed effect size.

In statistics, the difference between the value of the variable in the control group and that in the test drug group is known as effect size. This difference can be expressed as the absolute difference or the relative difference, e.g., in the weight loss example above, if the weight loss in the control group is 10 kg and in the test group it is 20 kg, the absolute effect size is 10 kg and the relative reduction with the test intervention is 10/20, or 50%.

We can estimate the effect size based on previously reported or preclinical studies. It is important to note that if the effect size is large between the study groups then the sample size required for the study is less and if the effect size between the study groups is small, the sample size required is large. In the case of observational studies, for example, if we want to find an association between smoking and lung cancer, since earlier studies have shown that there is a large effect size, a smaller sample would be needed to prove this effect. If on the other hand we want to find out the association between smoking and getting brain tumor, where the “effect” is unknown or small, the sample size required to detect an association would be larger.

## UNDERLYING EVENT RATE IN THE POPULATION

The underlying event rate of the condition under study (prevalence rate) in the population is extremely important while calculating the sample size. This unlike the level of significance and power is not selected by convention. Rather, it is estimated from previously reported studies. Sometimes it so happens that after a trial is initiated, the overall event rate proves to be unexpectedly low and the sample size may have to be adjusted, with all statistical precautions.

## STANDARD DEVIATION (SD OR Σ)

Standard deviation is the measure of dispersion or variability in the data. While calculating the sample size an investigator needs to anticipate the variation in the measures that are being studied. It is easy to understand why we would require a smaller sample if the population is more homogenous and therefore has a smaller variance or standard deviation. Suppose we are studying the effect of an intervention on the weight and consider a population with weights ranging from 45 to 100 kg. Naturally the standard deviation in this group will be great and we would need a larger sample size to detect a difference between interventions, else the difference between the two groups would be masked by the inherent difference between them because of the variance. If on the other hand, we were to take a sample from a population with weights between 80 and 100 kg we would naturally get a tighter and more homogenous group, thus reducing the standard deviation and therefore the sample size.

## SAMPLE SIZE CALCULATION

There are several methods used to calculate the sample size depending on the type of data or study design. The sample size is calculated using the following formula:

$$n\;=\;\frac{2{\left(Z_{a}+Z_{1–\beta }\right)}^{2_{\sigma }2_{,}}}{\Delta^{2}}$$

where *n* is the required sample size. For

Z_α_, *Z* is a constant (set by convention according to the accepted α error and whether it is a one-sided or two-sided effect) as shown below:

**Table:** 

α-error	5%	1%	0.1%
2-sided	1.96	2.5758	3.2905
1-sided	1.65	2.33	

For Z1-,β,Z is a constant set by convention according to power of the study as shown below:

**Table:** 

Power	80%	85%	90%	95%
Value	0.8416	1.0364	1.2816	1.6449

In the above-mentioned formula σ is the standard deviation (estimated) and Δ the difference in effect of two interventions which is required (estimated effect size).

This gives the number of sample per arm in a controlled clinical trial.

## EXAMPLE

This issue of the Journal has an article describing the benefits of ayurvedic treatment AyTP in patients of migraine in an open uncontrolled trial design.[3] If anyone wishes to confirm these results using a randomized controlled trial design where the effect of the ayurvedic intervention will be compared to standard of care in headache as measured by VAS how would we plan the sample size?

As seen above, we need the following values: *Z*_*α,*_ *Z*_1-β,_σ, standard deviation (estimated), and Δ, the difference in effect of two interventions. Let us assume we will accept a *p*<0.05 as acceptable and a study with 80% power; using the above tables, we get the following values: *Z*_α,_ is 1.96 (in this case we will be using a two-tailed test because the results could be bidirectional). *Z*_1-β,_ is 0.8416. The standard deviation (based on the data in the published paper) would be approximately 0.7. For Δ, the paper describes that the ayurvedic therapy has given a 35% effect. Previously it has been reported that sumatriptan at 50 mg improves headache by 50%.[4] Thus, the effect size would be 15% (i.e., 0.15).

The sample size for the new study will be

$$n=\frac{2{\left(1.96+0.8416\right)}^{2}{\left(0.72\right)}^{2}}{{\left(0.15\right)}^{2}}$$

= 362 per arm.

Calculating for a 10% drop-out rate one would need to complete approximately 400 patients per arm to be able to say with any degree of confi dence whether a difference exists between the two treatments.

## LIMITATIONS OF THE CALCULATED SAMPLE SIZE

The sample size calculated using the above formula is based on some conventions (Type I and II errors) and few assumptions (effect size and standard variation).

The sample size ALWAYS has to be calculated before initiating a study and as far as possible should not be changed during the study course.

The sample size calculation is also then influenced by a few practical issues, e.g., administrative issues and costs.

## References

[1] Kirby A, Gebski V, Keech AC (2002). Determining the sample size in a clinical trial. Med J Aust.

[2] Larsen S, Osnes M, Eidsaunet W, Sandvik L (1985). Factors influencing the sample size, exemplified by studies on gastroduodenal tolerability of drugs. Scand J Gastroenterol.

[3] Prakesh B, Babu SR, Sureshkumar K (2010). Response of Ayurvedic therapy in the treatment of migraine without aura. Int J Ayurveda Research.

[4] Cady RK, Sheftell F, Lipton RB, O'Quinn S, Jones M, Putnam G (2000). Effect of early intervention with sumatriptan on migraine pain: Retrospective analyses of data from three clinical trials. Clin Ther.

